# Relationship between plasma fibrinogen degradation products(FDP) and D-dimer levels and disease activity in rheumatoid arthritis: A STROBE compliant article

**DOI:** 10.1097/MD.0000000000030455

**Published:** 2022-09-09

**Authors:** FuYong Qiang, Hui Xu, Jun Sheng

**Affiliations:** a Department of Rheumatism and Immunology, The First Affiliated Hospital of Wannan Medical College, Anhui, China.

**Keywords:** C-reactive protein, D, -dimer, erythrocyte sedimentation rate, fibrinogen degradation products, rheumatoid arthritis

## Abstract

In this study, we aimed to investigate whether fibrinogen degradation products(FDP)and D-dimer could be used as serological indicators of rheumatoid arthritis(RA) activity, such as erythrocyte sedimentation rate (ESR), C-reactive protein (CRP), and platelets (PLT).

A total of 112 consecutive patients with RA between July 2018 and July 2020 were divided into moderate and high disease activity groups (disease activity score 28(DAS28) > 3.2, n = 60) and low disease activity and remission groups (DAS28≤3.2, n = 52). A total of 50 healthy volunteers were included in the control group, and FDP and D-dimer levels were compared across the three groups. The correlations of FDP and D-dimer levels with ESR, CRP, PLT, and DAS28 were analyzed. Analyses of the receiver operating characteristic(ROC) curves and area under the ROC curve (AUC) of FDP, D-dimer, ESR, CRP, and PLT levels were performed.

FDP and D-dimer levels were significantly higher in the high-activity compared to the low-activity and remission (*P* < .001), and the control (*P* < .001). No significant differences in FDP and D-dimer were observed between the low-activity and remission and the control (*P* > .05). FDP and D-dimer levels were positively correlated with ESR, CRP, PLT, and DAS28 (all *P* < .001). The ROC curves showed that the FDP and D-dimer levels could be used to evaluate the RA activity (all *P* < .001). The AUC of FDP was significantly larger than that of PLT (*P* = .047). FDP and D-dimer can be used as supplementary serological indicators to assess RA activity, in addition to ESR, CRP, and PLT.

## 1. Introduction

Rheumatoid arthritis (RA) is a systemic autoimmune disease in which patients present with symmetrical joint swelling and pain as its main clinical features. The erythrocyte sedimentation rate (ESR), C-reactive protein (CRP), and platelets (PLT) are commonly used as clinical laboratory indicators to reflect the activity of RA. The Disease Activity Score 28 (DAS28) is the most frequently used score to quantify disease activity. Currently, there is a lack of other convenient and effective serological indicators for assessing RA activity.^[1]^ Fibrinogen degradation products (FDP) and D-dimer are products of the activated fibrinolytic system that are closely associated with thrombotic diseases, and high FDP and D-dimer levels are also observed in patients with malignant diseases,^[2,3]^ pregnancy,^[4]^ prosthetic replacement arthroplasty,^[5]^novel coronavirus pneumonia,^[6]^and sepsis.^[7]^ Cerebral and myocardial infarctions are common thrombotic disorders associated with atherosclerosis. Studies have confirmed that the formation of atherosclerosis is related to systemic inflammation^[8–10]^and that there is a close relationship between the levels of FDP and D-dimer and atherosclerosis.^[11,12]^ These data suggest a strong correlation between FDP and D-dimer levels and systemic inflammation. Rheumatoid arthritis (RA) is a chronic systemic inflammatory disease. This study aimed to investigate the differences in FDP and D-dimer levels between patients with moderate and high disease activity and those with low disease activity and clinical remission. We also aimed to determine the correlations of FDP and D-dimer with ESR, CRP, PLT, and DAS-28, to provide an improved indication of RA activity.

## 2. Patients and Methods

### 2.1. Patients

This study analyzed 112 consecutive patients with RA between July 2018 and July 2020 were analyzed. The patients were divided into two groups based on the DAS28. Patients with DAS28>3.2 were included in the moderate and high disease activity groups, and patients with DAS28≤3.2 were included in the low disease activity and remission groups. Fifty age-matched, healthy volunteers were included in the control group. All RA patients conformed to the 2010 diagnostic criteria for RA established by the American Academy of Rheumatology/European Alliance Against Rheumatism (ACR/EULAR).^[13]^ The exclusion criteria were patients with tumors, systemic lupus erythematosus, ankylosing spondylitis, Sjogren’s syndrome, gout, liver, and kidney function abnormalities, a history of arteriovenous thrombosis, and those who had recently been prescribed anticoagulant and thrombolytic drugs. The study was approved by the institutional ethical committee(Ethics Committee of the First Affiliated Hospital of Wannan Medical College). All patients were recruited for the study after obtaining written informed consent.

### 2.2. Measurements of blood fibrinolysis and inflammatory indicators

FDP and D-dimer levels were determined using a Latex Immunoturbidimetry kit (STAGO automatic coagulation instrument, French) according to the manufacturer’s protocols. The standard detection ranges were 0–5 μg/mL for FDP and 0–0.5 μg/mL for D-dimer. Traditional inflammation indicators such as ESR, CRP, and PLT levels, were determined. The DAS28 score was used as previously described by Riel PL.^[14]^

### 2.3. Statistical analysis

Statistical analyses were performed using SPSS 26.0 and MedCalc 19.0 software. Nonparametric methods were used for all statistical comparisons, as all data were not uniformly distributed. Measurement data were represented by *M (P25, P75*), and the Wilcoxon Signed-Rank test was used for comparison among the groups. Spearman correlation (correlation coefficient *r*_*s*_) was used for the correlation analysis. The chi-square test was used to compare the ratios. ROC curves and AUC analyses were used to evaluate the sensitivity and specificity of all indicators. Statistical significance was set at *P* < .05.

## 3. Results

### 3.1. Comparison of the general conditions and inflammatory indicators in two groups

No significant differences in sex, age, or smoking were observed between the two groups (*P >* .05 for all). Swollen joint counts (SJC) (5.0(4.0, 7.0) vs 1.0(0.0, 1.0) (*Z* = 9.2, *P <* .001(95% CI: 4–5)), tender joint counts (TJC) (6.0(4.3, 8.0) vs 1.0(0.0, 1.0) (*Z* = 9.2, *P <* .001(95% CI:4–6)), morning stiffness (MS) (110.0(80.0, 150.0) vs 10.0(5.0,20.0) (*Z* = 9.1, *P <* .001(95% CI: 85–120)), ESR (66.0(30.5, 91.2) vs 14.0(8.0, 25.8) (*Z* = 7.1, *P <* .001(95% CI: 34–60)), CRP (40.5(19.0, 89.8) vs 2.0(1.0, 6.8) (*Z* = 8.2, *P <* .001(95%CI: 26–46)), RF (213.5(84.8, 400.5) vs 112.0(38.0, 232.8) (*Z* = 2.9, *P <* .001(95% CI: 24–159)), PLT (278.5(208.8, 358.0) vs 175.5(141.0, 202.8) (*Z* = 5.6, *P <* .001(95% CI: 71–137)), DAS28 (5.2(4.3,5.8) vs 10.01.6 (1.1,2.2) (*Z* = 9.1, *P <* .001(95% CI: 3.1–3.8)) were significantly higher in the moderate and high activity group compared to the low activity and remission group (Table 1).

**Table 1:** Comparison of the general conditions and inflammatory indicators in two groups.

	A(n=60)	B(n=52)	Ratio/Mediandifference(95%CI)	X^2^/Z	*P* value
Male/female	13/47	11/41	0.005(−0.147 to 0.157)	0.04	.95
Age (y)	55(49, 65)	54(49, 63)	1(−3 to 4)	0.34	.74
Smoking/No smoking	11/49	9/43	0.01(−0.132 to 0.152)	0.02	.89
SJC	5.0(4.0, 7.0)	1.0(0.0, 1.0)	5(4–5)	9.2	<.001
TJC	6.0(4.3, 8.0)	1.0(1.0, 1.0)	5(4–6)	9.2	<.001
MS (min)	110.0(80.0, 150.0)	10.0(5.0, 20.0)	100(85–120)	9.1	<.001
ESR (mm/h)	66.0(30.5, 91.2)	14.0(8.0, 25.8)	48(34–60)	7.1	<.001
CRP (mg/L)	40.5(19.0, 89.8)	2.0(1.0, 6.8)	37(26–46)	8.2	<.001
RF (IU/mL)	213.5(84.8, 400.5)	112.0(38.0, 232.8)	88.5(24–159)	2.9	.003
PTL (10^9^/L)	278.5(208.8, 358.0)	175.5(141.0,202.8)	104(71–137)	5.6	<.001
DAS28 score	5.2(4.3, 5.8)	1.6(1.1, 2.2)	3.4(3.1–3.8)	9.1	<.001

CRP = C-reactive protein, DAS28 = disease activity score 28, ESR = erythrocyte sedimentation rate, MS = morning stiffness, PLT = platelets, RF = rheumatoid factor, SJC = swollen joint counts, TJC = tender joint counts.

A= moderate and high activity group.

B= low activity and remission group.

### 3.2. Comparison of FDP and D-dimer in the patient groups

**Figure 1.**
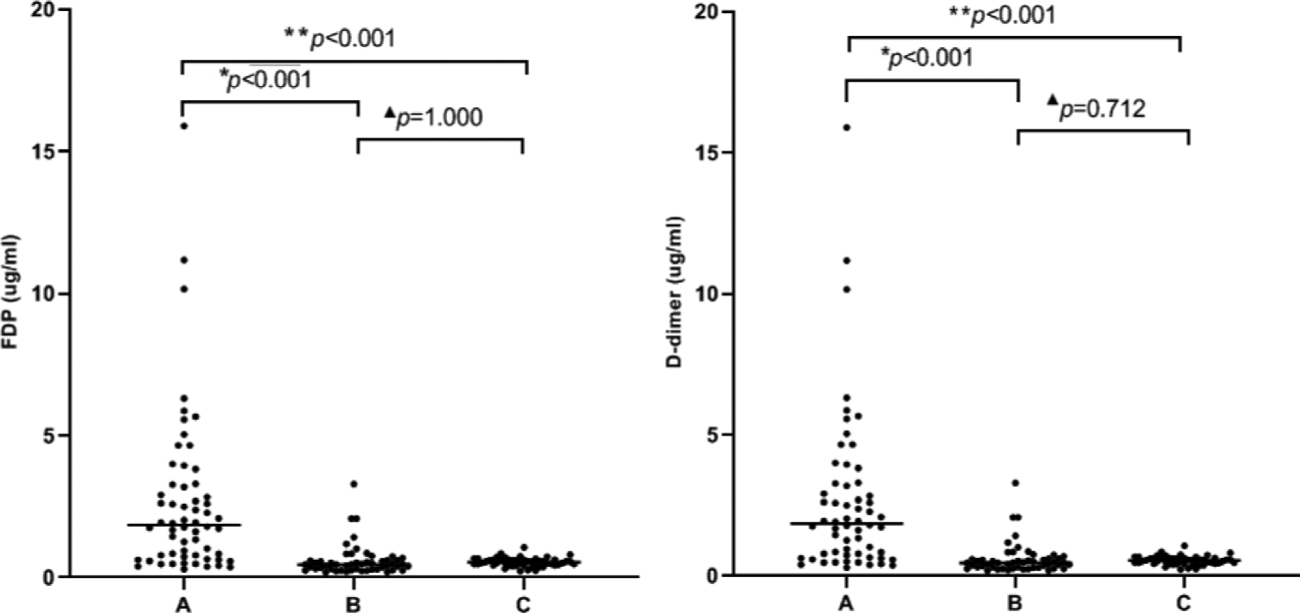
(A) Moderate and high activity group. (B) Low activity and remission group. (C) Healthy control group. **P < .001 compared with the healthy control group. *P < .001compared with the low activity and remission group. ^▲^*P >* .05 compared with the healthy control group.

The levels of FDP and D-dimer showed statistical abnormalities in the overall distribution in the three groups(FDP:*H* = 75.5, *P <* .001, D-dimer:*H* = 59.0, *P <* .001).The levels of FDP and D-dimer were significantly elevated in the moderate and high activity groups compared to the low activity and remission group (FDP: 7.7(3.6, 13.9) vs. 1.4(1, 1.9) (*P <* .001), D-dimer: 1.9(0.8, 3.3) vs 0.5(0.3, 0.6) (*P <* .001)) and the healthy controls (FDP:7.7(3.6, 13.9) vs 1.5(1, 1.8) (*P <* .001), D-dimer: 1.9(0.8, 3.3) vs 0.5(0.4, 0.6) (*P <* .001)). No significant differences in FDP and D-dimer were observed between the low activity and remission group and healthy control group (FDP:1.4(1, 1.9) vs 1.5(1, 1.8) (*P* = 1.000), D-dimer:0.5(0.3, 0.6) vs 0.5(0.4, 0.6) (*P* = .712)) (Fig. 1).

### 3.3. Correlation of FDP and D-dimer with ESR, CRP, PLT, and DAS28

Spearman’s correlation analysis showed that FDP and D-dimer levels were positively correlated with ESR(FDP:*r*_*s*_ = 0.63, *P <* .001; D-dimer: *r*_*s*_=0.67, *P <* .001), CRP(FDP:*r*_*s*_ = 0.65, *P <* .001; D-dimer:*r*_*s*_ = 0.71, *P <* .001), PLT(FDP:*r*_*s*_=0.41, *P <* .001; D-dimer:*r*_*s*_ = 0.43, *P <* .001), and DAS28(FDP:*r*_*s*_ = 0.69, *P <* .001; D-dimer: *r*_*s*_ = 0.70, *P <* .001) (Table 2).

**Table 2:** Correlation of FDP and D-dimer with ESR, CRP, PLT, and DAS28.

	ESR		CRP		PLT		DAS28	
	*r*_*s*_	*P* value	*r*_*s*_	*P* value	*r*_*s*_	*P* value	*r*_*s*_	*P* value
FDP	0.63	<.001	0.65	<.001	0.41	<.001	0.69	<.001
D-dimer	0.67	<.001	0.71	<.001	0.43	<.001	0.70	<.001

CRP = C-reactive protein; DAS28 = disease activity score 28; ESR = erythrocyte sedimentation rate; PLT = platelets.

Analyses of ROC curves and Comparisons of AUC of FDP and D-dimer with that of ESR, CRP, and PLT.

**Figure 2.**
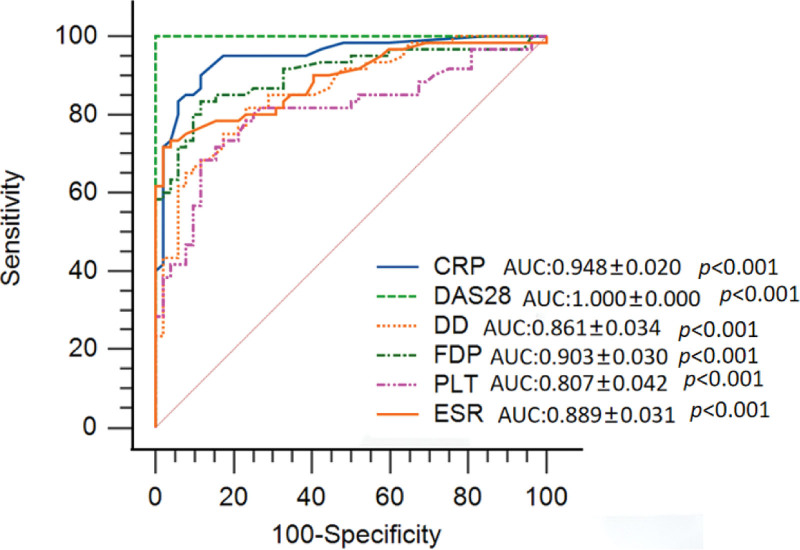
The ROC curves showed that FDP, D-dimer, ESR, CRP, and PLT could be used to evaluate RA disease activity (all *P <* .001).

The ROC curves showed that FDP, D-dimer, ESR, CRP, and PLT could be used to evaluate the activity of RA (all *P <* .001) (Fig. 2). The AUC of FDP(0.903 ± 0.030) was significantly larger than that of PLT(0.807 ± 0.042) (*Z* = 1.986, *P* = .047(95%CI: 0.001–0.191)). There were no differences among the AUC of D-dimer, ESR, and PLT*(P >* .05l) (Table 3).

**Table 3:** Comparisons of AUC of FDP and D-dimer with that of ESR, CRP, and PLT.

AUC	ESR(0.889 ± 0.031)		CRP(0.948 ± 0.020)		PLT(0.807 ± 0.042)	
	Mean difference (95%CI)	(*Z, P*)	Mean difference	(*Z, P*)	Mean difference	(*Z, P*)
FDP(0.903 ± 0.030)	0.013(−0.058 to 0.086)	(0.376, .706)	−0.046(−0.017 to 0.108)	(1.429, .153)	0.096(0.001–0.191)	(1.986, .047)*
D-dimer(0.861 ± 0.034)	−0.028(−0.047 to 0.100)	(0.793, .459)	−0.088(0.023–0.151)	(2.680, .007)	0.054(−0.045 to 0.154)	(1.063, .288)

AUC = area under roc curve, CRP = C-reactive protein, ESR = erythrocyte sedimentation rate, FDP = fibrinogen degradation products, PLT = platelets.

* The AUC of FDP was significantly more compared to that of PLT (*Z* = 1.986, *P* = .047)

## 4. Discussion

The initiation and development of RA are mediated by a systemic inflammatory response. ESR is often associated with elevated levels of globulins including RF but lacks specificity. CRP is the most common acute-phase protein that can be elevated in the early stages of RA and can promote the proliferation of synovial fibroblasts in the joints of RA patients.^[15]^CRP can activate the RANKL system and directly induce osteoclast activation aggravating bone destruction.^[16]^ Platelets are involved in the coagulation process and in the immune and inflammatory responses that can amplify joint inflammation in RA.^[17]^ Although most patients with RA have elevated ESR, CPR, and PLT levels during the active phase, these changes do not occur in all patients. There is a need for improved indicators that can be used to evaluate RA activity and to guide clinical diagnosis and treatment. Coagulation is closely associated with inflammation,^[18]^ and the test of FDP and D-dimer is convenient, rapid, and cheap. Therefore, FDP and D-dimer may be indicators for rapid assessment of RA activity.

The ROC curve analyses indicated that FDP and D-dimer could be used as serological indicators to evaluate the activity of RA, such as ESR, CRP, and PLT. However, AUCs analyses showed that FDP and D-dimer were not advantageous compared to ESR, CRP, and PLT. Therefore, we consider that FDP and D-dimer could be supplementary indicators to assess the activity of RA in addition to ESR, CRP, and PLT. FDP is the degradation product of fibrinogen. D-dimer is derived from the cross-linked fibrin clot dissolved by plasmin and reflects the fibrinolytic function. Elevated levels of FDP and D-dimer reflect a hypercoagulable state and secondary fibrinolytic activity enhancement. Siegfried^[19]^showed that D-dimer and fibrinogen levels were significantly increased in active juvenile RA patients. In this study, the levels of FDP, D-dimer, ESR, CRP, and PLT were significantly higher in the high-activity group than in the low-activity and the remission groups. FDP and D-dimer levels are mainly correlated with ESR, CRP, PLT, and DAS28. This phenomenon indicates a hypercoagulable state and microthrombosis in patients with moderately or highly active RA. This may be due to the presence of many inflammatory cytokines, including TNF-**α**and IL-6, which are released into the plasma during highly active RA. TNF-**α**causes damage to the vascular endothelium^[20]^ and significantly inhibits the growth of vascular endothelial cells.^[21]^These observations show that TNF-**α**can damage vascular endothelial cells to trigger the exposure of endothelial collagen and activation of the endogenous coagulation pathway. These processes can lead to microthrombosis. IL-6 induces endothelial cells to produce plasminogen activator inhibitor-1^[22]^ and can also enhance the adhesion of vascular endothelial cells^[23]^making the body more susceptible to hypercoagulation. Furthermore, RF is an autoantibody with an Fc segment of denatured IgG as the target antigen, which is significantly increased in the serum during active RA. RF can directly combine with IgG to form immune complexes and is deposited on the vascular endothelium. This deposition leads to vasculitis by activating the complement system and triggering inflammatory reactions in vascular endothelial cells. Watanabe S^[24]^found that the condition of RF-positive patients was more serious in those patients with ANCA-related vasculitis without RA indicating that RF may be involved in the occurrence of vasculitis. High titers of IgG-RF and IgM-RF are likely to lead to rheumatoid vasculitis.^[25,26]^ Higher positive values and titers of RF in RA patients with rheumatoid vasculitis than in RA patients without rheumatoid vasculitis.^[27]^ The occurrence of rheumatoid vasculitis allows for increased exposure of endothelial collagen, which can further activate the endogenous coagulation pathway, leading to local thickening of the vascular intima and stenosis of the lumen. These changes alter the blood rheology and facilitate the formation of a mural thrombus. In addition, local inflammation of the joint allows fibrinogen to penetrate the joint cavity and deposit on the synovial surface.^[28]^ Fibrinogen and its degradation products in the joint cavity can promote cytokine activation and exacerbate local inflammation.^[29]^ These mechanisms indicate that high cytokine levels, high titers of RF, and increased capillary permeability of joints are typically observed in patients with highly active RA. In these patients, the vascular endothelium is more easily damaged and rheumatoid vasculitis can occur more easily, which activates the endogenous coagulation pathway causing hypercoagulability and microthrombosis. Similarly, secondary activation of the fibrinolytic system leads to increased FDP and D-dimer levels.

In summary, the main serological indicators of active RA are ESR, CRP,and PLT levels. FDP and D-dimer levels provide a new perspective for determining disease activity. Increased levels of FDP and D-dimer are more conducive to evaluating RA disease activity, particularly when RA is active but is not associated with high levels of ESR and CRP. FDP and D-dimer are effective as supportive indicators in addition to traditional indicators of RA.

However, the sample size was too small in this study and a cross-sectional study could not accurately reflect the relationship between the FDP and D-dimer levels and RA disease activity. Therefore, we need to expand the sample size and formulate a more rigorous clinical design to clarify the relationship between the FDP and D-dimer levels and RA disease activity in subsequent studies.

## Author contributions

Conceptualization: FuYong Qiang, Jun Sheng.

Data curation: Hui Xu.

Formal analysis: Jun Sheng.

Investigation: Hui Xu.

Methodology: Jun Sheng.

Software: FuYong Qiang.

Supervision: Jun Sheng.

Writing—original draft:FuYong Qiang.

Writing—review & editing: Jun Sheng.

Approval of final manuscript: all authors.
